# Practical Quantum Private Database Queries Based on Passive Round-Robin Differential Phase-shift Quantum Key Distribution

**DOI:** 10.1038/srep31738

**Published:** 2016-08-19

**Authors:** Jian Li, Yu-Guang Yang, Xiu-Bo Chen, Yi-Hua Zhou, Wei-Min Shi

**Affiliations:** 1 School of Computer, Beijing University of Posts and Telecommunications, Beijing 100876, China; 2 College of Computer Science and Technology, Beijing University of Technology, Beijing 100124, China; 3 Information Security Center, State Key Laboratory of Networking and Switching Technology, Beijing University of Posts and Telecommunications, Beijing, 100876, China

## Abstract

A novel quantum private database query protocol is proposed, based on passive round-robin differential phase-shift quantum key distribution. Compared with previous quantum private database query protocols, the present protocol has the following unique merits: (i) the user Alice can obtain one and only one key bit so that both the efficiency and security of the present protocol can be ensured, and (ii) it does not require to change the length difference of the two arms in a Mach-Zehnder interferometer and just chooses two pulses passively to interfere with so that it is much simpler and more practical. The present protocol is also proved to be secure in terms of the user security and database security.

According to the credibility of participants, quantum cryptographic applications are mainly divided into two categories: quantum cryptographic protocols with trusted parties and the ones with distrusted parties. In the first category, participants are trusted and the threats are mainly from outside attackers. The most famous example is the quantum key distribution (QKD) protocol where a secure identical key is shared by two remote parties so that an adversary, Eve, cannot obtain any information about the key (up to a small failure probability). In contrast, in the second category, the main security threats come from the inside participants who maybe are dishonest. The main applications include quantum secret sharing (QSS), quantum Byzantium agreement (QBA), quantum private comparison (QPC), quantum oblivious transfer (QOT), quantum private database query (QPDQ), etc.

This letter focuses on the second category, in particular, the QPDQ protocol. In QPDQ protocols, the user Alice wants to obtain an item in Bob’s database without leaking which item she wants (user security), and Bob does not want Alice to get any information about other items (database security). The problem has been formalized as symmetrically private information retrieval (SPIR)^1^ by Gerther *et al*. Because unconditionally secure SPIR is impossible^2^, cheat-sensitive SPIR protocols are desirable. The term “cheat-sensitive” means that dishonest Bob will run the risk of being discovered if he tries to obtain the address queried by Alice.

As the quantum counterpart of the SPIR problem, QPDQ^3^,^4^,^5^,^6^,^7^ has attracted lots of attention. Giovannetti *et al*. presented the first QPDQ protocol (GLM protocol)^4^. Subsequently, Olejnik presented an improvement of GLM protocol^7^. In the above QPDQ protocols^4^,^5^,^6^,^7^, the database is modeled by an oracle operation. However, its practical implementation is limited by the high dimension of the oracle operation. To solve this problem, Jakobi *et al*.^8^ suggested the first practical QPDQ protocol (J-protocol) based on the SARG04 QKD protocol^9^. Since then, the QKD-based QPDQ model has attracted a great deal of attention and many theoretical and experimental attempts have been made at devising QKD-based QPDQ protocols^10^,^11^,^12^,^13^,^14^,^15^,^16^,^17^,^18^,^19^,^20^,^21^,^22^,^23^,^24^.

Existing QKD-based QPDQ protocols generally involve three stages: (i) quantum oblivious key distribution (QOKD), (ii) classical post-processing (CPP) of the generated oblivious key, and (iii) classical private query (CPQ). The goal of QOKD is to help the user Alice and the database holder Bob to share a raw key in such a way that it is known wholly to Bob and a fraction to Alice. The CPP of the generated oblivious key is aimed to reduce Alice’s known bits in the final key to roughly one bit thereby ensuring the database security. The CPP algorithm of the oblivious key is of vital importance to the efficiency and security of QPDQ protocols. Some CPP algorithms have been proposed^8^,^25^,^26^. For example, J-protocol gave a *kN* → *N* method, that is, it transforms a raw key with length *kN* into an *N*-bit final key, which requires a communication complexity of Ο(*N*log*N*) (here *N* is the total number of items in the database)^8^. To further reduce this communication complexity, Rao *et al*. presented two improved methods of J-protocol, i.e., *N* → *N* and *rM* → *N* ones so that the communication complexity is reduced to Ο(*N*)^25^. Here, *r* and *M* are integers and satisfy the constraint: *rM* ≪ *N*. Unfortunately, the reduction in communication complexity comes at the cost that the parity information about the final key bits is elicited by Alice^27^. To solve the linear correlation among the adjacent final bits, Yang *et al*.^26^ proposed a nonlinear CPP scheme for QKD-based QPDQs.

It is shown that both the QOKD and CPP algorithms determine the efficiency and security of QPDQ protocols. Inspired by a recently proposed round-robin differential phase-shift (RRDPS) QKD protocol^28^ utilizing single-photon signal of multiple optical pulses, Liu *et al*.^22^ proposed a novel QPDQ protocol. Different from previous QKD-based QPDQ protocols, in their protocol, the number of the items an honest user will obtain is always one and the failure probability is always zero. Therefore, no CPP process is required and the efficiency and security of the QPDQ protocol^22^ can be improved.

However, the original RRDPS QKD protocol^28^ requires to change the length difference of the two arms in a Mach-Zehnder interferometer (MZI). Actually it is difficult to change the length difference of the two arms in a MZI with high speed using the current technology. To solve this problem, Guan *et al*.^29^ proposed an alternative scheme, i.e., the passive round-robin differential phase-shift (PRRDPS) QKD scheme which does not require to change the length difference of the two arms in a MZI and just chooses two pulses passively to interfere with so that it is much simpler and more practical. Inspired by the PRRDPS QKD scheme^29^, in this paper, we propose a novel QPDQ protocol. The proposed protocol is more practical than the work by Liu *et al*.^22^ and actually is its extension.

## Results

### The PRRDPS QKD protocol

Let’s first review the original RRDPS QKD protocol proposed by Sasaki *et al*.^28^. In the original RRDPS QKD protocol^28^, the sender Alice selects *s*_*i*_ ∈ {0, 1} to denote a random phase from {0, *π*} and encodes it on the *i*th one of *L* pulses. For each incoming *L*-pulse block, the receiver Bob implements a single-photon interference with a MZI (see Fig. 2 of ref. 28). Concretely, Bob first splits each pulse by a half beam-splitter and then randomly adjusts the length difference between the two arms of the MZI, ranging from 1 to *L* − 1 pulse periods. Then the pulses in the two light paths pass the second beam-splitter. After getting a detection click, Bob identifies which two pulses interfere and then announces the corresponding pulse indices *i*, *j* to Alice. Alice and Bob can take the relative phase between these two pulses as the raw key, i.e., *s*_*A*_ = *s*_*B*_ = *s*_*i*_ ⊕ *s*_*j*_, where *s*_*i*_ ∈ {0, 1} denotes the phase of Alice’s *i*th pulse.

In the original RRDPS QKD protocol^28^, Bob has to adjust the length difference between the two arms of the MZI randomly. However, it is difficult to change the length difference of the two arms in a MZI with high speed using the current technology. To solve this problem, Guan *et al*.^29^ proposed an alternative scheme where two pulses are chosen passively to interfere with. When Bob receives a pulse block from Alice, he prepares a local *L*-pulse reference encoded at phase 0. This *L*-pulse reference interferes with the *L*-pulse signal sent by Alice on a beam splitter, as shown in Fig. 1(c) of ref. 29. For each block, Bob records the status of his two detectors with timestamps, *i* and *j*.

Assume both Alice and Bob have exactly one photon in their respective *L*-pulse trains. The states of Alice and Bob can be represented by


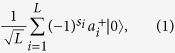



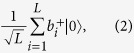


respectively, where 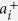, 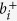 are the creation operators. Since there is a photon in each *L*-pulse block from Alice and Bob respectively, Bob would obtain at most two detection clicks. He chooses the block where there are exactly two detection clicks and announces their positions *i* and *j* (if *i* = *j*, the detection result is discarded). The raw key is the relative phase between these two pulses in the *L*-pulse signal. Alice can derive this phase difference from her record. After the interference and Bob’s post-selection, the quantum state at the two detectors becomes one of the following states:


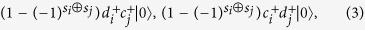



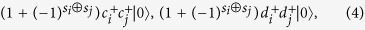


where 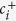 and 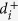 are the creation operators at the two detectors respectively, as shown in Fig. 1(c) of ref. 29. This means if Alice’s *i*th and *j*th pulses have the same phase, i.e., *s*_*i*_ = *s*_*j*_, the two clicks should be triggered by the same detector. If Alice’s *i*th and *j*th pulses have different phases, the two clicks should be triggered by different detectors. Thus Bob can derive the relative phase by comparing the measurement results of the *i*th and *j*th pulses.

### The QPDQ protocol based on the PRRDPS QKD protocol

Without loss of generality, the proposed protocol involves two parties: the database holder Bob and the user Alice. It includes two stages: (1) the QOKD stage, and (2) the CPQ stage. The details of the proposed QPDQ protocol are described as follows.

#### The QOKD stage

(1) Alice and Bob each have exactly one photon in their *N* + 1-pulse trains. The states of Bob and Alice can be represented by


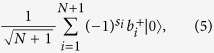



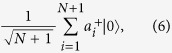


respectively, where *s*_*i*_ ∈ {0, 1} denotes the phase of Bob’s *i*th pulse.

(2) Alice chooses the block where there are exactly two detection clicks. After the interference and Alice’s post-selection, the quantum state at the two detectors becomes one of the four states in Eqs (3) and (4) (see Fig. 1(c) of ref. 29). Alice announces the position *i* (if *i* = *j*, the detection result is discarded). Alice can derive this phase difference from her record and obtain the key bit *s*_*i*_ ⊕ *s*_*j*_. Then Bob takes the bits *s*_*B*_ = *s*_*i*_ ⊕ *s*_1_, *s*_*i*_ ⊕ *s*_2_, …, *s*_*i*_ ⊕ *s*_*i*−1_, *s*_*i*_ ⊕ *s*_*i* + 1_, …, *s*_*i*_ ⊕ *s*_*N*+1_ as his *N*-bit key, *K*.

#### The CPQ stage

Bob encrypts his database with the key *K*. Suppose Alice knows the *j*th bit *K*_*j*_ and wants to retrieve the *i*th item *X*_*i*_. She publishes the number *s* = *j* − *i*. Then Bob shifts *K* by *s* and uses the new key to encrypt his database. Because *X*_*i*_ is in fact encrypted by *K*_*j*_, Alice can decrypt *X*_*i*_ correctly.

### Security analysis

In the QPDQ protocols, the security is mainly analyzed in terms of the user security and database security.

### Database security

Assume Alice is dishonest. When Alice cheats, Bob is automatically regarded as honest. If Alice wants to obtain more items in Bob’s database, she has to try to obtain more key bits in the raw key *s*_*B*_. In the ideal case, according to Eq. (7), after the QOKD stage, Alice obtains the relative phase between the two pulses in the *N* + 1-pulse signal and thus obtains one and only one key bit *s*_*i*_ ⊕ *s*_*j*_. Different from ref. 11, where Alice who takes charge of preparing the photon signal maybe prepares a fake signal to cheat Bob, in the present scheme, the server Bob is responsible for preparing the pulse signal and he should prepare the ideal *N* + 1-pulse single-photon signal.

However, in the practice case, Bob’s pulse train maybe has 2 ≤ *n* ≤ *N* + 1 photons and Alice’s pulse train maybe has 2 ≤ *m* ≤ *N* + 1 photons. With this assumption, there are three cases. The first one is that both clicks come from Bob’s photons. The second one is that both clicks come from Alice’s photons. The third one is that one click is from Bob’s photon and the other is from Alice’s photon. In this case, although Bob and Alice may have multiple photons, but only one of his and her photons is respectively selected so that this case is identical to the ideal single-photon case. Here, we focus on the first two cases.

### Both clicks come from Bob’s photons

Assume the two photons are both from Bob, then after the interference by the beam splitter which replaces 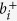 by 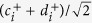 and 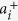 by 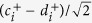, the resulting detection result will be


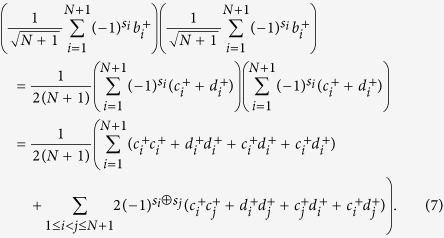


For the first item in Eq. (7), Alice discards it in the post-selection because *i* = *j.* One can see that the events that the same detector clicks (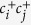 and 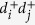) and different detector clicks (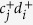 and 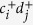) have the same amplitude and thus the same probability. Thus whatever Bob’s phase encoding is, there will be 50% bit error for Alice’s bit.

### Both clicks come from Alice’s photons

For this case, the situation is similar. Assume the two photons are both from Alice, then the resulting detection result will be


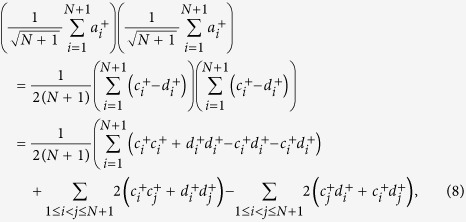


and the same conclusion holds.

### One click is from Bob’s photon and the other is from Alice’s photon

Assume the two photons are from Bob and Alice, respectively. After the interference by the beam splitter, then the resulting detection result becomes


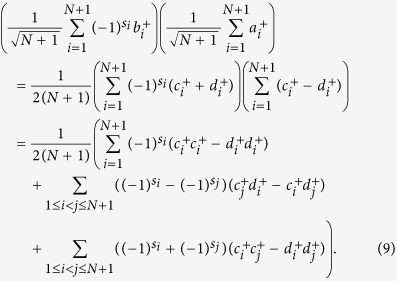


If *s*_*i*_ ⊕ *s*_*j*_ = 1, the detectors on different sides click. Otherwise, the detector on the same side clicks twice. Thus, Alice announces the position *i*. The raw key is the relative phase between these two pulses in the *N* + 1-pulse signal. Alice can derive this phase difference from her record and obtain the key bit *s*_*i*_ ⊕ *s*_*j*_. Then Bob takes the bits *s*_*B*_ = *s*_*i*_ ⊕ *s*_1_, *s*_*i*_ ⊕ *s*_2_, …, *s*_*i*_ ⊕ *s*_*i*−1_, *s*_*i*_ ⊕ *s*_*i*+1_, …, *s*_*i*_ ⊕ *s*_*N*+1_ as his *N*-bit key, *K*.

Even if Alice obtains clicks in multiple positions, she can only announce a position *i*. Her attack cannot bring her any benefit. Therefore, one can see that to realize the proposed QPDQ protocol, Alice and Bob should prepare the ideal *N* + 1-pulse single-photon state.

### User security

Assume Bob is dishonest. When Bob cheats, Alice is automatically regarded as honest. As we know, unconditionally secure private queries are known to be impossible, so cheat-sensitive QPQ is desirable^5^,^7^,^8^,^10^,^11^,^12^,^13^,^14^,^15^,^16^,^17^,^18^,^19^,^20^,^21^,^22^,^23^,^24^. Our protocol is also cheat-sensitive in terms of the user security. This is because Bob cannot obtain the retrieval address and give a correct answer for the query simultaneously. Next we will briefly discuss the probability with which Bob can obtain the position of Alice’s bit in the raw key. Now let’s discuss two cases.

### Bob takes a fake *N* + 1-pulse-single-photon-signal attack

Assume dishonest Bob’s state can be represented by a general state 
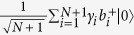
. Here 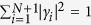.

After the interference by the beam splitter, the resulting detection result becomes


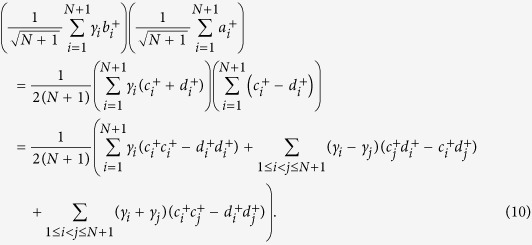


Alice obtains the result with two clicks (*i*, *j*) on the same side and different sides with probabilities 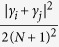 and 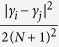, respectively. This implies that Bob can bias the probability of obtaining results for Alice. But this does not decrease Alice’s privacy. In this sense it is still cheat-sensitive. Bob will inevitably lose the knowledge about the value of the key bit when he tries to obtain its conclusiveness. As a result, Bob might send a wrong answer to Alice, which will be found by Alice at a later time.

### Bob takes a fake *N* + 1-pulse-two-photon-entanglement-signal attack

Dishonest Bob maybe takes another cheating strategy. Assume Bob prepares a two-photon entangled state in his *N* + 1-pulse train. Bob keeps one photon and sends the other photon to Alice. After Bob’s *N* + 1-pulse signal interferes with Alice’s *N* + 1-pulse reference on the beam splitter, Bob can determine the position of the corresponding entangled photon by measuring the partner photon kept by him to know which position it collapses at after interference. This cheating strategy cannot be found by Alice. However, Bob only can determine which position the corresponding entangled photon collapses at without obtaining any information about which position Alice’s photon collapses at. Because the positions of the detector clicks decide Alice’s key bit. That is, if the detectors click on different sides, Alice judges *s*_*i*_ ⊕ *s*_*j*_ = 1 and her key bit is 1. Otherwise, *s*_*i*_ ⊕ *s*_*j*_ = 0 and her key bit is 0. Therefore, if Bob’s entangled photon is positioned at the timestamp *i*, and Alice’s announcement is also *i*, Bob cannot obtain the information about Alice’s bit. However, if Bob’s entangled photon is positioned at the timestamp *j*, and Alice’s announcement is *i*, Bob will obtain the information about Alice’s bit. Therefore, Bob can obtain Alice’s bit with a probability 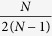. To reduce the probability, Alice can take some eavesdropping detection strategies before interference on the beam splitter. For example, Alice receives the *N* + 1-pulse signal and attains the total photon number *n* of the *N* + 1-pulse train by performing a photon number quantum nondemolition (QND) measurement. If the pulse train contains more than one photon (*n* > 1), Alice can judge that Bob is cheating and abort the protocol. Otherwise, she continues the protocol.

## Discussion

Let’s recall the original RRDPS QKD protocol^28^. Zhang *et al*.^30^ found that the phase error rate bound given in the original RRDPS protocol based on weak coherent pulses (WCPs)^28^ is not tight, since the phase error rate should never exceed 1/2. Then they developed a tighter bound for the phase error rate by using phase-randomization and the decoy-state method^31^,^32^. More importantly, in the original RRDPS QKD protocol^28^, the length of the pulse block, *L*, is a critical parameter which affects the performance and technical difficulty of implementation of the protocol. It has also been proved by Zhang *et al*.^30^ that, with new analysis method with and without decoy states, and also by the decoy-state method, even if *L* is small (i.e. *L* = 32), the performances is very close to the optimal *L* case.

In contrast, in the PRRDPS QKD protocol^29^, the optimal *L* is more than 8000, which is not practical. So our proposed QPDQ protocol based on the PRRDPS QKD protocol^29^ will be more practical and of performance improvement if Zhang *et al*.’s results^30^ are introduced into the proposed QPDQ protocol. That is, we may use the decoy-state method in our proposed protocol so that it is more practical and easier to implement in practice.

## Additional Information

**How to cite this article**: Li, J. *et al*. Practical Quantum Private Database Queries Based on Passive Round-Robin Differential Phase-shift Quantum Key Distribution. *Sci. Rep.* **6**, 31738; doi: 10.1038/srep31738 (2016).
